# Benign-Appearing Palmar Lesion in a Young Woman: The Importance of Considering Digital Papillary Adenocarcinoma

**DOI:** 10.7759/cureus.108350

**Published:** 2026-05-06

**Authors:** Eva Cannaferina, Mathias Rouveyrol, Audrey Michot, Lucie Peuvrel, Ludovic Ardouin

**Affiliations:** 1 Plastic Surgery, Hand Surgery, Centre Hospitalier Universitaire (CHU) de Bordeaux, Bordeaux, FRA; 2 Hand Surgery Department, Clinique Mutualiste de Bretagne Occidentale, Quimper, FRA; 3 Reconstructive and Oncologic Department, Bergonié Institute, Bordeaux, FRA; 4 Dermatology Department, Institut de Cancérologie de l'Ouest (ICO) Cancer Center, Saint Herblain, FRA; 5 Hand Surgery Department, Institut de la main Nantes-Atlantique, Saint-Herblain, FRA

**Keywords:** adnexal tumors, caraderm, digital papillary adenocarcinoma, hpv-42, slow-mohs

## Abstract

Adnexal tumors are rare skin tumors, representing less than one percent of cutaneous cancers, with diagnosis and management remaining complex due to the absence of a consensual clinical, histological, and prognostic classification.

Among them, digital papillary adenocarcinoma is an exceptional malignant eccrine tumor responsible for local recurrences and metastatic dissemination. Its clinical presentation is nonspecific, which can lead to diagnostic delays. Classically described in middle-aged men and localized on the fingers, it can nevertheless present under misleading clinical and topographical forms.

We report the atypical case of a 22-year-old female patient who, since childhood, had a painless palmar lesion that later became sensitive and was initially interpreted as a benign lesion. The diagnosis was only made after excision and histological confirmation following a review by the national Rare Cancers in Dermatology network (CARADERM). Management involved surgical re-excision with exhaustive analysis of margins using the Slow-Mohs technique combined with sentinel lymph node biopsy, without evidence of residual tumor. After a prolonged follow-up of 79 months, no local or distant recurrence was observed.

This case highlights the importance for hand surgeons to be aware of this type of tumor and for pathologists to rely on a double review by the national network in cases of clinico-pathological doubt to optimize diagnosis and management.

## Introduction

Adnexal carcinomas are rare malignant cutaneous tumors arising from sweat glands, hair follicles, or sebaceous glands, representing less than one percent of all skin cancers [1].

Digital papillary adenocarcinoma (DPA) is an uncommon and aggressive adnexal tumor of eccrine origin, with an estimated incidence of 0.08-0.10 per 1,000,000 individuals per year [2,3]. It may arise de novo or from malignant transformation of a benign adnexal lesion. Diagnosis is often difficult due to the absence of a specific clinical presentation [4]. The disease course may be indolent and painless, mimicking a benign lesion; however, local recurrence occurs in up to 50% of cases, and 14-41% of patients develop metastases, most frequently involving the lungs [4].

DPA typically affects men (male-to-female ratio 4:1) between 40 and 70 years old, although pediatric cases have been reported. The upper limb is the most common site (81%), particularly the pulp or periungual regions of the fingers (80%), followed by the toes. Despite its designation, atypical locations such as the forearm, vulva, and perianal region have been described [3,4].

Clinically, DPA presents as a nodular or papular lesion, sometimes ulcerated or painful, with a mean size of 1.7-2 cm (range 0.4-4.3 cm) [3].

Histologically, it arises in the dermis and may extend to deeper structures, including subcutaneous tissue, muscle, or bone. Its morphology may mimic metastatic adenocarcinomas or other adnexal tumors. A strong association with HPV 42 has been recently identified, suggesting a potential diagnostic marker and oncogenic role [1,5].

Atypical location and indolent progression may lead to misdiagnosis and delayed treatment.

## Case presentation

Clinical presentation

A 22-year-old woman presented with a palpable, supra-aponeurotic subcutaneous nodule on the left hypothenar eminence, evolving asymptomatically for approximately 15 years. No sensory symptoms were reported.

**Figure 1:**
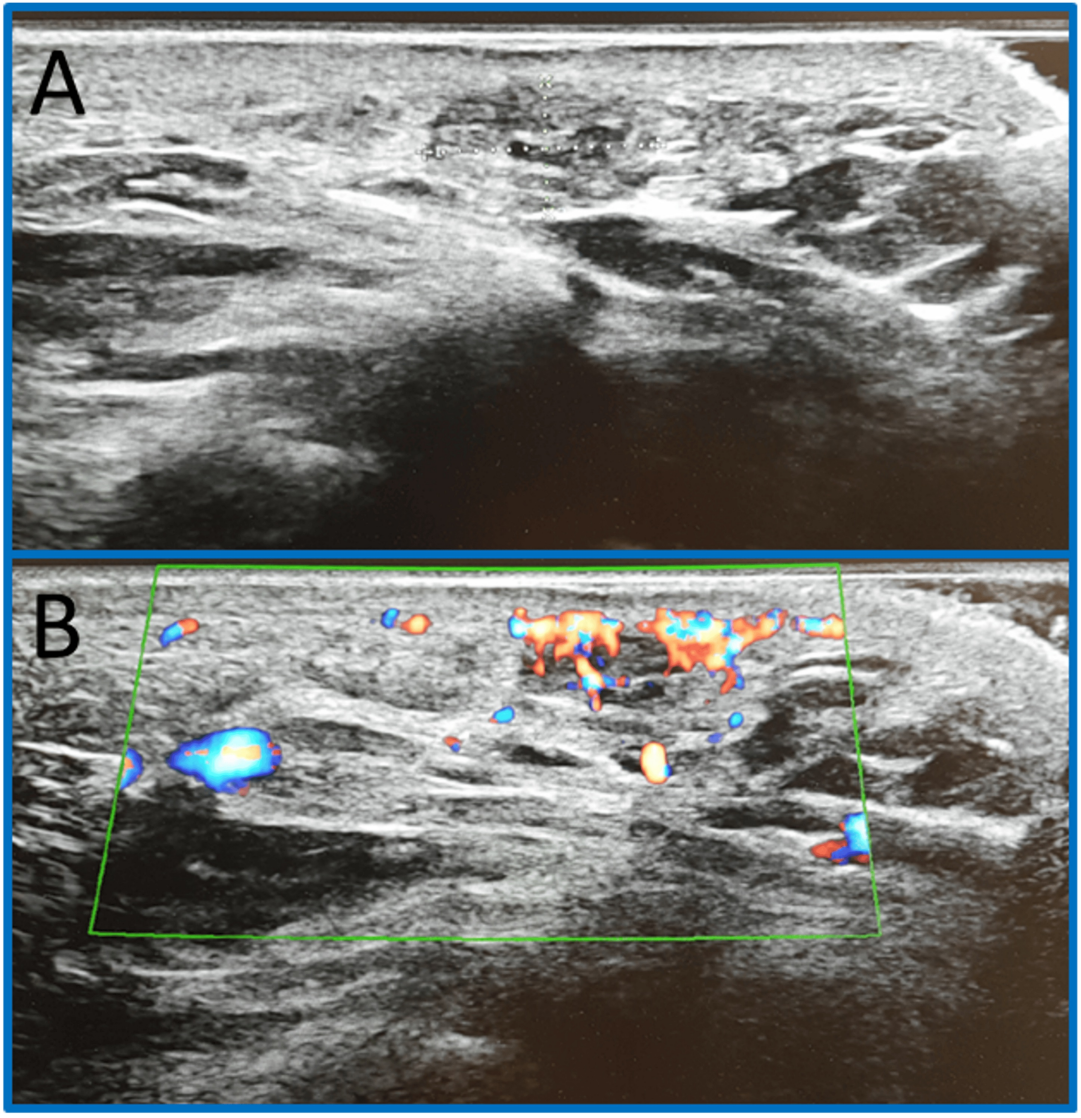
Lesion on longitudinal ultrasound (A) with Doppler showing venous flow (B), suggestive of hemangioma

Ultrasound, performed after the beginning of mild discomfort, revealed a well-defined subcentimeter lesion (7 × 5.5 × 4 mm), suggestive of a hemangioma (Figure 1). The lesion exhibited a spongiform echotexture with compressible serpiginous vascular structures and a venous Doppler signal within surrounding adipose tissue.

Given its benign appearance, surgical excision was performed without prior biopsy, additional imaging, or planned safety margins.

Histopathological findings

Intraoperatively, the lesion was well circumscribed and surrounded by adipose tissue.

**Figure 2:**
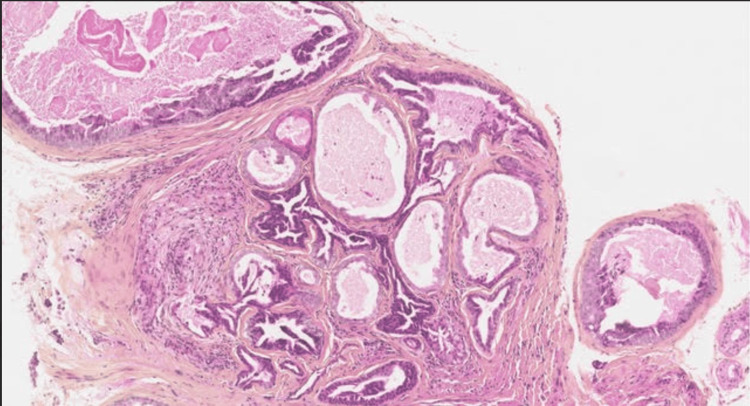
Hematoxylin and eosin-stained sections showing a dermal multilobular tumor with focal cystic components and papillary structures

Histological examination revealed fibro-adipose tissue containing Pacinian corpuscles, dissected by ductal structures of variable size. Some were cystic and filled with necrotic material, while others formed clustered configurations. The lesion was lined by cuboidal and columnar epithelial cells forming papillary projections. Cells showed eosinophilic cytoplasm, oval nuclei, and prominent nucleoli. No perineural invasion or vascular emboli were identified (Figure 2).

These findings were consistent with digital papillary adenocarcinoma and were confirmed after expert review within the French Rare Skin Cancer Network (CARADERM).

Management and follow-up

**Figure 3:**
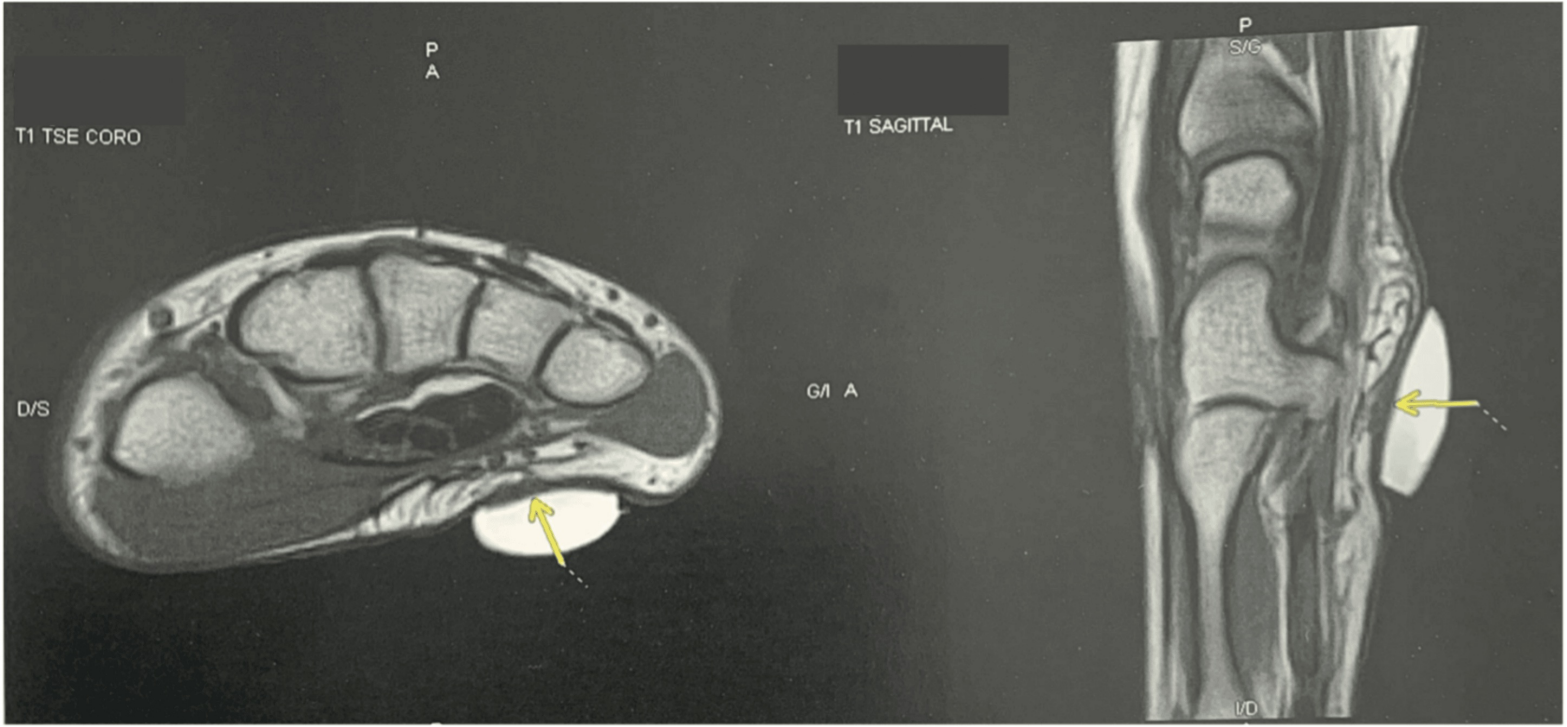
Two-month postoperative MRI The yellow arrow indicates scar tissue measuring 10 × 6 × 3 mm

Post-diagnostic staging included MRI of the wrist and hand at two months, which showed no local recurrence, and a thoraco-abdomino-pelvic CT scan, which was normal (Figure 3).

Following multidisciplinary discussion, re-excision was performed three months later with 10 mm margins, combined with slow-Mohs histological analysis and sentinel lymph node biopsy. Excision extended to the palmar aponeurosis, and reconstruction was achieved using a full-thickness skin graft from the inner part of the left arm. All specimens, including the sentinel lymph node, were negative.

Follow-up consisted of clinical examination and ultrasound every three months, with CT imaging every six months for three years, followed by semiannual clinical follow-up for two years and then self-monitoring. Long-term surveillance was recommended due to the risk of late recurrence.

After 79 months of follow-up, no recurrence or metastasis has been observed.

## Discussion

Diagnostic challenge

DPA is a rare tumor with significant diagnostic challenges, especially in atypical presentations mimicking benign lesions. Awareness among clinicians is essential, despite the absence of standardized guidelines.

These lesions may be mistaken for various benign or malignant conditions, including cysts, glomus tumors, pyogenic granulomas, giant cell tumors, hidradenomas, squamous cell carcinoma, or infections.

In the present case, clinical and imaging findings suggested a benign vascular lesion, leading to initial management without photography, biopsy, or margin-controlled excision.

Although most commonly located on the fingers, DPA may also occur on the hand, wrist, toes, or atypical sites such as the forearm or anogenital region [2,4,6-11]. Bone involvement has also been described [12]. Unusual clinical presentations (recurrent paronychia [9], felon [13]) may delay diagnosis.

Therapeutic management

As adnexal tumors may arise de novo or from pre-existing benign lesions, excision of any benign adnexal lesion is recommended.

There is currently no consensus regarding optimal treatment, including wide local excision, Mohs micrographic surgery (MMS), and amputation.

MMS offers complete margin assessment and tissue preservation, making it particularly suitable for functionally sensitive areas such as the hand. Some studies suggest lower recurrence rates with MMS [2] compared to conventional excision, while others found no significant differences [14], although data remain limited.

The most important prognostic factor appears to be the time to complete excision with negative margins. Delays beyond six months are associated with significantly increased risks of recurrence, metastasis, and mortality [14].

Margins of approximately 1 cm are generally recommended and may be extended in more aggressive cases [14].

The role of sentinel lymph node biopsy remains debated. While it may provide prognostic information, its impact on survival is unclear [4,14-16]. None of the patients with negative lymph node biopsies had a recurrence or metastasis during the follow-up, which could lead to using lymph node biopsy as a prognostic factor.

Given the potential for late recurrence and metastasis, prolonged follow-up is recommended, which could follow melanoma guidelines [12].

Recently, analysis of the CARADERM database by Zagala et al. [17] enabled characterization of the profile of these lesions in order to establish a prognostic classification. The study included 979 patients with a minimum follow-up of three years. Regarding digital papillary adenocarcinoma, the overall risk of recurrence and the metastatic risk were classified as low. This analysis calls into question the presumed aggressiveness of these lesions, whose natural history can only be clarified through improved knowledge, supported by comprehensive case collection.

Contribution of molecular biology

Specialized networks such as CARADERM in France provide valuable support in diagnostically challenging cases through expert histopathological review and multidisciplinary discussion [5].

Molecular biology has improved diagnostic accuracy. A strong association between DPA and HPV 42 has been demonstrated, with viral DNA detected in most cases and absent in other adnexal tumors. This finding supports its role as a diagnostic marker and suggests a potential oncogenic mechanism [18,19]. Moreover, DNA methylation profiling may aid in differentiating DPA from other adnexal neoplasms [18].

## Conclusions

This case highlights the clinical variability of digital papillary adenocarcinoma and the risk of misdiagnosis in atypical presentations. Due to its rarity, its natural history remains incompletely understood, and management is not standardized. However, early complete excision with clear margins is essential to reduce recurrence and metastatic risk. This observation supports a proactive surgical approach to soft tissue masses of the hand, even when minimally symptomatic. Improved knowledge will depend on prospective data collection and long-term follow-up. Expert networks such as CARADERM play a key role in enhancing diagnosis, management, and understanding of this rare tumor.
